# Increased serum anti-ceramide antibodies and decreased sphingosine-1-phosphate levels in patients with obstructive sleep apnea syndrome as potential markers of endothelial dysfunction

**DOI:** 10.3389/fmolb.2025.1644828

**Published:** 2025-09-03

**Authors:** Andrzej Wiśniewski, Elżbieta Wiśniewska, Łukasz Lewandowski, Izabela Nowak, Monika Kosacka

**Affiliations:** ^1^ Laboratory of Immunogenetics and Tissue Immunology, Hirszfeld Institute of Immunology and Experimental Therapy Polish Academy of Sciences, Wrocław, Poland; ^2^ Department of Pulmonology and Lung Oncology, Wrocław Medical University, Wrocław, Poland

**Keywords:** obstructive sleep apnea syndrome, sphingosine 1-phosphate, anti-ceramide antibodies, ceramide, sphingolipid rheostat, endothelial dysfunction

## Abstract

**Objective:**

Sphingosine-1-phosphate (S1P) and ceramide are bioactive sphingolipids that have been associated with some obstructive sleep apnea (OSA) comorbidities like coronary artery disease (CAD), insulin resistance, diabetes mellitus, hypertension, cardiac dysfunction, and ischemic stroke. On the other hand, S1P and ceramide play key roles in maintaining endothelial homeostasis, which is impaired by repetitive hypoxia/reoxygenation and sleep fragmentation characteristic of OSA. Since the exact role of S1P and ceramide in OSA is still poorly explored, the present study aimed to compare the levels of S1P and anti-ceramide antibodies (ceramide-Ab) in OSA patients and controls.

**Methods:**

We recruited 153 subjects (104 patients and 49 controls). The concentrations of anti-ceramide antibodies and S1P were measured using the ELISA technique.

**Results:**

We detected significantly higher levels of anti-ceramide antibodies in the OSA group than in the control group (median 318.0 vs. 247.7 ng/mL, p < 0.0001). By contrast, S1P levels were markedly higher in the controls than in the OSA patients (median 1,006.0 vs. 573.9 ng/mL, p < 0.0001). No correlation was observed between either ceramide-Ab or S1P concentrations and the following variables: OSA severity (AHI), desaturation index (DI), BMI, average SaO_2_, minimum SaO_2_, and C-reactive protein (CRP). Additionally, we noted a positive correlation between BMI and AHI (Spearman r = 0.5051, p < 0.0001), as well as between BMI and DI (Spearman r = 0.55, p < 0.0001). Conversely, BMI negatively correlated with mean SaO_2_ (Spearman r = - 0.58, p < 0.0001) and with minimum SaO_2_ (Spearman r = - 0.44, p < 0.0001). A middle-strong positive correlation was observed between BMI and serum level of CRP (Spearman r = 0.60, p < 0.0001).

**Conclusion:**

We demonstrated that anti-ceramide antibody levels were significantly increased, whereas S1P levels were decreased in patients with obstructive sleep apnea in comparison to healthy subjects. These results suggest that the balance between ceramide and S1P (known as sphingolipid rheostat) may be dysregulated in the course of OSA. We suggest that ceramide-Ab might become a valuable positive biomarker of the disease with S1P as a negative biomarker.

## 1 Introduction

Obstructive sleep apnea (OSA) is a common sleep disorder characterized by repetitive partial or complete collapse of the upper airway during sleep, resulting in apnea or hypopnea (Kohler and Stradling, 2010). This leads to many consequences, the most important of which are intermittent hypoxia and arousals from sleep causing sleep fragmentation (Lv et al., 2023; Osman et al., 2018). OSA is commonly associated with a wide range of cardiovascular diseases (CVD), including coronary artery disease (CAD), hypertension, heart failure, arrhythmia, stroke, and pulmonary hypertension (Li and Ren, 2022). The main pathomechanisms that contribute to the elevated cardiovascular risk in sleep apnea syndrome include chronic activation of the sympathetic nervous system, oxidative stress, chronic inflammation and endothelial dysfunction (Unnikrishnan et al., 2015; Javaheri et al., 2017; Harańczyk et al., 2022; Peracaula et al., 2022). It has been demonstrated that repetitive hypoxia/reoxygenation cycles and sleep fragmentation impair endothelial function. In particular, in OSA, endothelial nitric oxide production and repair capacity are restricted, whereas oxidative stress and inflammation are intensified (Atkeson et al., 2009).

Sphingolipids are both structural components in the plasma membranes of eukaryotic cells and signaling molecules regulating a variety of biological functions (Sasset and Di Lorenzo, 2022). Proper sphingolipid metabolism is crucial for maintaining endothelial cell homeostasis (Lai et al., 2022). Of all sphingolipids, ceramide and sphingosine-1-phosphate (S1P) in particular are able to differentially regulate cellular functions by modulating opposing signaling pathways. In this context, the mutual, dynamic balance of these two interconnected lipid mediators has been termed the ceramide/S1P rheostat (Piccoli et al., 2023).

Ceramides are a family of bioactive sphingolipids acting as second messengers in cell signaling pathways. They can activate various kinases and transcription factors, leading to the regulation of cell growth, proliferation, differentiation, and apoptosis (Shen et al., 2025). Endothelium (Cantalupo et al., 2020), hepatocytes (Merrill et al., 1995) and adipose tissue (Akawi et al., 2021) are the sources of circulating ceramides. Elevated plasma concentrations of ceramides have been associated with multiple risk factors for coronary artery disease (Poss et al., 2020), obesity (Haus et al., 2009), diabetes mellitus (Fretts et al., 2020), hypertension (Spijkers et al., 2011), as well as cardiac remodeling and dysfunction (Ji et al., 2017). Moreover, circulating ceramides positively correlate with systemic insulin resistance and inflammation (Haus et al., 2009; de Mello VD et al., 2009). Interestingly, ceramide levels are notably higher within atherosclerotic plaques (Schissel et al., 1996), which may contribute to atherosclerosis by promoting the infiltration of low-density lipoproteins (LDLs) into the endothelium and their aggregation within the intima of artery walls (Meeusen et al., 2020).

S1P is a potent lipid mediator that regulates various physiological as well as pathological processes in the vasculature and immune system (Winkler et al., 2015). This important molecule is produced intracellularly from ceramide. Firstly, ceramide is converted to sphingosine by ceramidase, and then sphingosine is phosphorylated into S1P by sphingosine kinase (SphK) 1 or 2 (Kurano and Yatomi, 2018). After extracellular release, S1P exerts pleiotropic effects through binding to specific G protein-coupled receptors S1PR1-5 (Liu et al., 2012). These five receptors are expressed on various cell types from the immune, respiratory, cardiovascular, hepatic, and neurological system (Thuy et al., 2014). Through differential binding to its receptors, S1P regulates many physiological and pathological processes including, blood pressure (Intapad, 2019), vascular endothelial function (Weigel et al. 2023), atherosclerosis (Soltau et al., 2016), coagulation and inflammation (Obinata and Hla, 2012). S1P is detected at high concentrations in plasma where it is bound mainly to high-density-lipoprotein (HDL) via apolipoprotein M (ApoM), or to albumin (Christoffersen et al., 2011). The main sources of S1P in circulation are erythrocytes, platelets, and the endothelium (Kurano and Yatomi, 2018). With regards to endothelium dysfunction (a known complication in OSA), *in vitro* studies have shown that sphingosine-1-phosphate exerts a protective effect against endothelial cell damage induced by hypoxemia (Yu et al., 2018).

The roles of bioactive sphingolipids, including the most well studied ones - ceramide and S1P, remain poorly understood in the context of obstructive sleep apnea. Until now, only two reports have been published on this subject. In the first, Lebkuchen et al. (2018) in a comprehensive metabolomic analysis, described a modest increase in the concentration of certain ceramides (d18:1/24:4) in OSA male patients. In the second study, Horváth et al., 2023 reported a significantly increased concentration of anti-ceramide antibodies (ceramide-Ab) as well as S1P in OSA patients compared with healthy controls. Considering the abovementioned reports, we sought to investigate whether serum S1P and ceramide-Ab are altered in our well characterized cohort of OSA patients and whether they are associated with OSA severity, specific clinical parameters, and comorbidities such as coronary heart disease, diabetes mellitus and hypertension. Additionally, we intended to explore whether S1P and ceramide-Ab could serve as reliable biomarkers for the diagnosis of OSA and the assessment of disease severity.

## 2 Materials and methods

### 2.1 Study design

One hundred and four newly diagnosed OSA patients (77 men and 27 women) and 49 healthy controls (42 men and 7 women) were qualified for the study. All tested participants came from Poland. The median age of the patients was 60.5 (30–80) years and the median apnea/hypopnea index (AHI) was 33.7 (Table 1). In the examined group, the majority consisted of patients with severe OSA (AHI>30/hour) - 60 patients (median AHI 61.2/hour). There were also 28 patients with moderate OSA (AHI 15–30/hour, median AHI 20.7/hour) and 15 patients with mild OSA (AHI 5–15/hour, median AHI 9.9/hour). The following cardiovascular diseases coexisted with OSA: hypertension in 73 patients (70.2%), diabetes in 28 (27%), coronary heart disease in 26 (25%) and 3 had undergone a stroke (2.8%). Moreover, 7 patients had COPD (6.7%). All the patients received standard treatment for comorbidities.

**TABLE 1:** Clinical characteristics of all research participants.

Characteristics	Patients N = 104	Controls N = 49
Sex, Male (%)	77 (74)	42 (85.7)
Median age/min-max	60.5/30–80	42/27–75
Smoking currently, n (%)	24 (23.1)	34 (69.4)
Smoking in the past, n (%)	61 (58.7)	42 (85.7)
Median BMI/min-max	34.2/21.6–60.2	
Median AHI/min-max	33.7/3.2–107.9	
Median DI/min-max	36.15/0–144.8	
Average SaO_2_	93	
Minimum SaO_2_	76	
Median Glucose/min-max	104/78–316	
Median CRP/min-max	2.34/0.6–126	
Ceramide-Ab concentration (ng/mL)/min-max	318/103.8–1,182	247.7/54.16–470.7
S1P concentration (ng/mL)/min-max	573.9/265.4–1,168	1,006/357–1,429

BMI, body mass index; AHI, apnea-hypopnea index; DI, desaturation index; SaO2, saturation; S1P, sphingosine-1-phoshate.

#### 2.1.1 Polysomnography

All patients underwent a nocturnal polysomnography using the Alice 6 LDe Polysomnographic Sleep System (Philips Respironics). During 8 h of nocturnal sleep, the following parameters were measured: airflow with the use of oronasal thermal sensor and nasal pressure sensor, chest and abdomen movements, body position, snoring, oxygen saturation using a finger clip sensor, and sleep stages. According to the standard criteria of the American Academy of Sleep Medicine (AASM): apnea is defined as a reduction in the peak signal excursion by ≥ 90% of the pre-event baseline for more than 10 s and hypopnea as a reduction in airflow by at least 30% of the pre-event baseline using nasal pressure accompanied by either a ≥3% arterial oxygen desaturation or an arousal (Berry et al., 2012). In all cases manual scoring was carried out after automatic scoring. The following parameters were used in the diagnosis of OSA and the severity assessment: AHI, oxygen desaturation index - ODI, mean arterial oxygen saturation (SaO_2_) during sleep, and minimum SaO_2_ at the end of sleep apnea/hypopnea episodes.

The control group consisted of healthy blood donors without any chronic diseases including obstructive sleep apnea. The median age was 42 (27–75) (Table 1).

This study was carried out according to the Declaration of Helsinki and accepted by the Ethics Committee of Wrocław Medical University (No. 217/2024). All cases and controls signed written informed consent to participate in the study.

### 2.2 ELISA measurements

Six ml of venous blood was collected into BD Vacutainer tubes with a clot activator (Becton Dickinson). After 30 min of clotting at room temperature (RT), the samples were centrifuged (1500 RPM for 10 min in RT), aliquoted, and stored at −70 °C for further analysis. The EH2564 Human S1P (Sphingosine 1 Phosphate) ELISA Kit (FineTest) and MBS3804520 Human Ceramide antibody (ceramide-Ab) ELISA Kit (MyBioSource) were used to determine the serum levels of S1P and ceramide-Ab, respectively. Serum samples were diluted eight-fold for Ab-ceramide and five-fold for S1P. All samples were tested in duplicate and the average values were used in the analysis. The concentration of the sphingolipids was calculated based on standard curves provided with the kits, and results were expressed in ng/mL. Optical density was determined at a wavelength of 450 nm using an Infinite F50 microplate reader (Tecan Trading AG, Switzerland).

### 2.3 Statistical analysis

The D'Agostino-Pearson K2 normality test was used to determine whether the data deviated from the Gaussian distribution. As the data were not normally distributed, nonparametric Mann-Whitney or Kruskal–Wallis tests were utilized. Data are presented as medians with ranges (minimum and maximum values). Correlations between serum S1P and ceramide-Ab levels with selected clinical parameters including BMI, AHI, DI, average and minimum saturation, glucose concentration and CRP were analyzed using Spearman’s rank correlation test. A bivariate analysis using the Mann-Whitney test was utilized to assess the association between ceramide-Ab or S1P concentrations and the presence of comorbidities such as coronary heart disease, diabetes, and hypertension. Medians for S1P and ceramide-Ab concentrations were compared between subgroups of patients who were positive or negative for a given comorbidity. Due to the limited number of patients with COPD and stroke, such calculations for these conditions were not possible. All of the abovementioned statistical analyses were performed in GraphPad Prism ver. software 5.0 (San Diego, CA, United States). The power of Mann-Whitney tests for statistically significant results was calculated using G*Power software ver.3.1.9.7. Finally, two multiple linear regression models were applied to examine the combined influence of several independent variables: *OSA*, comorbidities (*hypertension, diabetes, CHD, COPD, stroke*) as well as *age* and *female sex* on the dependent variable - *ceramide-Ab* (Model 1) or *S1P* (Model 2) concentrations. Input data for S1P and ceramide-Ab levels were transformed using the Box-Cox transformation to make their distribution more normal. DATAtab (online statistics calculator) was used to create multiple linear regression models. A p value <0.05 was considered significant.

## 3 Results

### 3.1 Ceramide antibody and S1P levels

**FIGURE 1:**
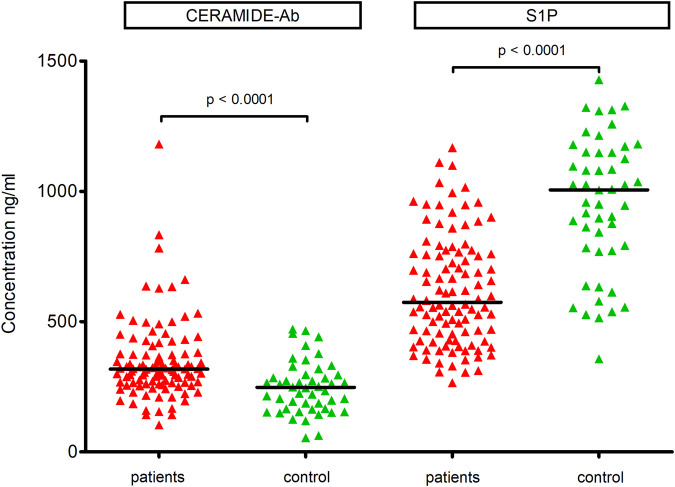
Comparison of median concentrations of ceramide-Ab and S1P between OSA patients and controls.

We observed a significantly higher level of anti-ceramide antibodies in the OSA group than in the control group (median 318.0 vs. 247.7, p < 0.0001, Figure 1). The power for this test achieved 98% (effect size d = 0.77, α error = 0.05, sample size for patients = 104 and for controls = 48). Additionally, we observed no correlation between ceramide-Ab concentration and BMI (p = 0.56), AHI (p = 0.43), average SaO_2_ (p = 0.37), minimum SaO_2_ (p = 0.85), CRP (p = 0.11) and fasting glucose (p = 0.25). However, a trend towards significance was noted for the DI parameter (p = 0.06).

The S1P level was significantly higher in controls than in OSA patients (median 1,006.0 vs. 573.9 ng/mL, p < 0.0001, Figure 1). The power for this test achieved 100% (effect size d = 1.41, α error = 0.05, sample size for patients = 104 and 49 for controls). Similar to ceramide-Ab, the concentration of S1P did not correlate with BMI (p = 0.40), AHI (p = 0.78), average SaO_2_ (p = 0.95), minimum SaO_2_ (p = 0.56), DI (p = 0.88), CRP (p = 0.97), or fasting glucose (p = 0.15).

Bivariate analysis of subgroups revealed no correlation between ceramide-Ab or S1P levels and the presence of comorbidities, including coronary heart disease (ceramide-Ab, p = 0.59; S1P, p = 0.31), diabetes (ceramide-Ab, p = 0.36; S1P, p = 0.69), and hypertension (ceramide-Ab, p = 0.45; S1P, p = 0.88).

**FIGURE 2:**
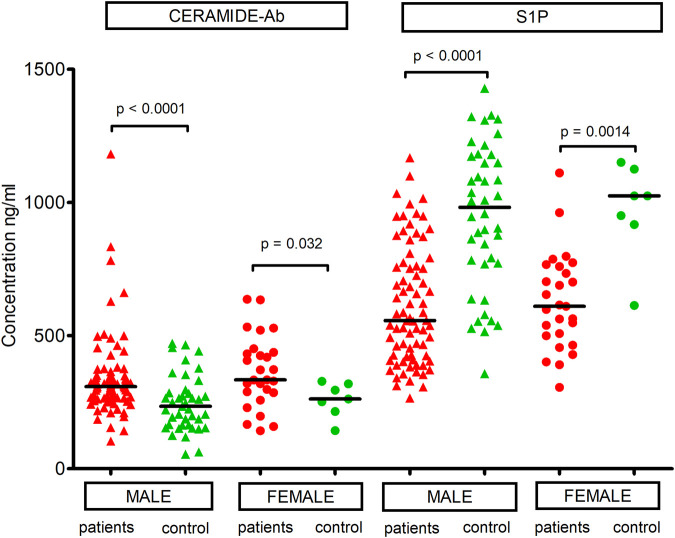
Comparison of median concentrations of ceramide-Ab and S1P between male patients and male controls, as well as between female patients and female controls.

Due to the low number of females in our control group (N = 7), we were not able to precisely determine whether the concentrations of the two studied sphingolipids were associated with sex. Nevertheless, bivariate analysis revealed that ceramide-Ab and S1P concentrations were significantly higher in male patients compared to male controls, as well as in female patients compared to female controls (Figure 2). In summary, the influence of sex on ceramide-Ab and S1P levels was not demonstrated by the analysis in subgroups.

Cigarette smoking among patients and controls did not influence the results obtained. Anti-ceramide antibodies concentrations were significantly higher both in smoking patients vs. smoking controls (313.9 vs. 256.2 ng/mL; p = 0.0017) and in non-smoking patients vs. non-smoking controls (319.5 vs. 224.8 ng/mL; p = 0.0025). In the case of S1P, its concentration was also significantly higher both in smoking controls vs. smoking patients (1,031 vs. 541.7 ng/mL; p < 0.0001) and in non-smoking controls vs. non-smoking patients (904 vs. 586.7 ng/mL; p < 0.0001). For the calculations above, both those who had never smoked and those who had smoked previously but were not current smokers were classified as non-smokers.

Two multiple linear regression models were used to estimate the independent correlation of ceramide-Ab (Model 1) and S1P (Model 2) concentrations with several variables: *OSA, hypertension, diabetes, CHD, COPD, stroke* as well as *age* and *female sex* (Table 2, 3). Model 1 and Model 2 were statistically significant (df = 8, F = 3.55, p = 0.001, and df = 8, F = 7.9, p < 0.001, respectively). In both models, the independent variable *OSA* significantly impacted ceramide-Ab levels (β = 0.41, SE = 0.27, p < 0.001) and S1P levels (β = - 0.54, SE = 0.3, p < 0.001). None of the other tested independent variables had influence on the concentrations of either sphingolipid.

**TABLE 2:** Multiple linear regression model performed to examine the influence of the independent variables: *OSA, hypertension, diabetes, CHD, COPD, stroke, age* and *female sex* on the dependent variable - *ceramide-Ab concentrations.*

	Unstandardized Coefficients	Standardized Coefficients				95% confidence interval for B	
Model	B	Beta	Standard error	t	p	lower bound	upper bound
*Constant*	9.18		0.41	22.38	<0.001	8.37	9.99
*OSA*	1.05	0.41	0.27	3.88	<0.001	0.51	1.58
*Hypertension*	−0.26	−0.11	0.26	−1.02	0.31	−0.78	0.25
*Diabetes*	0.43	0.14	0.29	1.48	0.14	−0.14	1.01
*CHD*	−0.34	−0.11	0.29	−1.19	0.236	−0.91	0.23
*COPD*	0.49	0.09	0.46	1.06	0.291	−0.43	1.41
*Stroke*	−0.31	−0.04	0.72	−0.43	0.67	−1.72	1.11
*Age*	0	0.01	0.01	0.07	0.944	−0.02	0.02
*Female sex*	0.2	0.07	0.23	0.86	0.393	−0.26	0.66

**TABLE 3:** A multiple linear regression model performed to examine the influence of the independent variables: *OSA, hypertension, diabetes, CHD, COPD, stroke, age* and *female sex* on the dependent variable - *S1P concentrations.*

	Unstandardized Coefficients	Standardized Coefficients				95% confidence interval for B	
Model	B	Beta	Standard error	t	p	Lower bound	Upper bound
*Constant*	14.73		0.45	32.69	<0.001	13.84	15.62
*OSA*	−1.65	−0.54	0.3	−5.56	<0.001	−2.24	−1.07
*Hypertension*	0.13	0.05	0.29	0.45	0.65	−0.44	0.7
*Diabetes*	−0.09	−0.02	0.32	−0.27	0.788	−0.72	0.55
*CHD*	−0.3	−0.08	0.32	−0.94	0.35	−0.93	0.33
*COPD*	0.32	0.05	0.51	0.63	0.529	−0.69	1.34
*Stroke*	0.39	0.04	0.79	0.49	0.623	−1.18	1.95
*Age*	−0	−0.04	0.01	−0.41	0.682	−0.02	0.01
*Female sex*	0.14	0.04	0.26	0.54	0.587	−0.37	0.65

### 3.2 Association between BMI and selected clinical parameters

**FIGURE 3:**
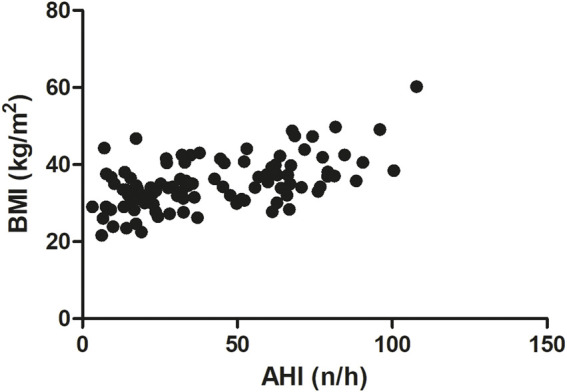
Correlation between OSA severity (AHI) and BMI (Spearman r = 0.50, p < 0.0001); n - number of apneas and hypopneas that occur per hour of sleep.

**FIGURE 4:**
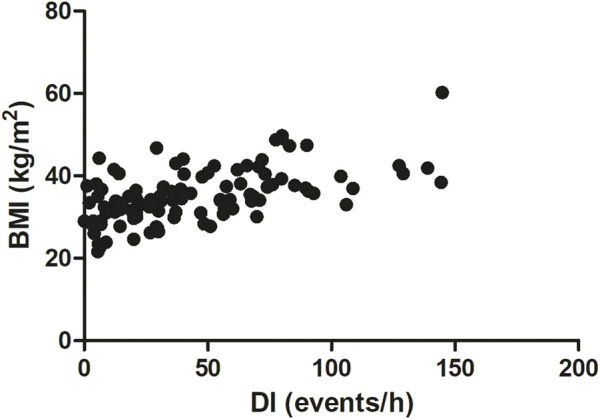
Correlation between desaturation index (DI) and BMI (Spearman r = 0.55, p < 0.0001).

**FIGURE 5:**
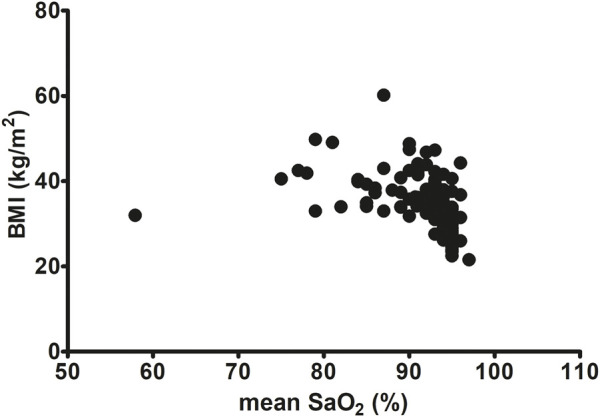
Correlation between mean SaO_2_ and BMI (Spearman r = - 0.58, p < 0.0001).

**FIGURE 6:**
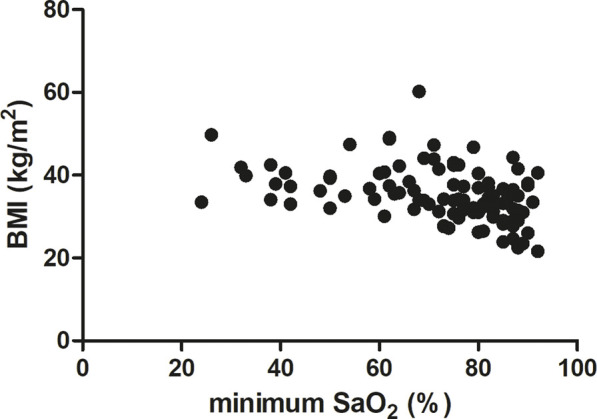
Correlation between minimum SaO_2_ and BMI (Spearman r = - 0.44, p < 0.0001).

In our study BMI was significantly correlated with OSA severity (AHI). We observed a middle-strong positive correlation between BMI and AHI (Spearman r = 0.5051, p < 0.0001, Figure 3). A similar correlation was noted between BMI and DI (Spearman r = 0.55, p < 0.0001, Figure 4). On the other hand, we detected a middle-strong but negative correlation between BMI and mean SaO_2_ (Spearman r = - 0.58, p < 0.0001, Figure 5) and minimum SaO_2_ (Spearman r = - 0.44, p < 0.0001, Figure 6).

**FIGURE 7:**
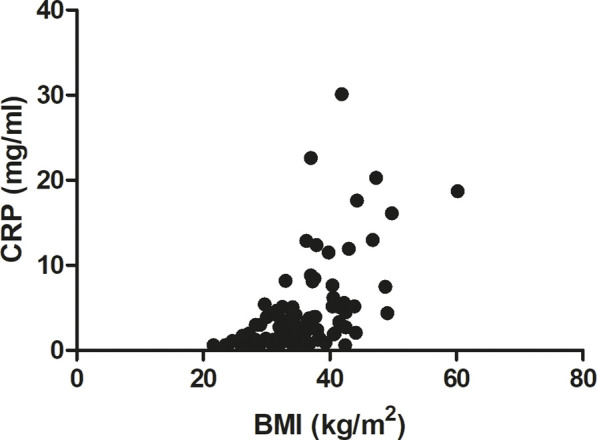
Correlation between BMI and CRP (Spearman r = 0.60, p < 0.0001).

Additionally, a middle-strong positive correlation was also observed between BMI and serum CRP levels (Spearman r = 0.60, p < 0.0001, Figure 7), whereas a weak positive correlation was found with fasting glucose (Spearman r = 0.20, p = 0.04 data not shown).

## 4 Discussion

The primary aim of this study was to evaluate the concentrations of two key sphingolipids, sphingosine-1-phosphate and ceramide in serum samples collected from OSA patients and healthy individuals. Instead, however, in order to detect ceramide we decided to measure the level of anti-ceramide antibodies. We believe that their levels simply correspond to the levels of ceramides present in the blood (higher levels of ceramide-Ab likely reflect higher levels of ceramide). There are several publications in which the authors evaluated antibodies against ceramides using the ELISA technique in relation to diseases, such as leprosy (Singh et al., 2010), peripheral neuropathies (Sykam et al., 2017), non-small cell like cancer (Bűdi et al., 2025), and finally in OSA (Horváth et al., 2023). To the best of our knowledge, 2 years ago during the time of planning our study, no commercially available ELISA test for ceramide was available. Therefore instead of using an ELISA test, ceramides were often evaluated by more sophisticated and sensitive techniques such as liquid chromatography in connection with mass spectrometry (LC-MS/MS). Unfortunately, we were unable to apply them. Therefore, we decided to use an indirect method of ceramide assessment (by detecting ceramide-Ab) in the form of an ELISA test.

In our study, ceramide-Ab levels were significantly increased in OSA patients compared to controls. A very similar result was reported by Horváth et al. (2023) although they noted a larger difference in antibody concentration between patients and controls (∼4-fold). In this report, it was 1.3-fold. As mentioned in the introduction, elevated levels of specific ceramides (d18:1/24:4) were also reported in male OSA patients in comprehensive lipidomic analysis (Lebkuchen et al., 2018). This indirectly indicates that the level of ceramide-Ab may actually reflect the amount of ceramide in circulation in the case of OSA. As also mentioned in the introduction, increased ceramide levels have been reported in several conditions frequently accompanying obstructive sleep apnea, such as obesity (Haus et al., 2009), coronary artery disease (Poss et al., 2020), insulin resistance (Boon et al., 2013), diabetes (Fretts et al., 2020), hypertension (Spijkers et al., 2011) or heart failure (Ji et al., 2017). Moreover, some studies showed that specific plasma ceramide ratios, including C24:0/C16:0 and C22:0/C16:0, independently correlate with major adverse cardiovascular events in patients with and without coronary artery diseases (Laaksonen et al., 2016).

In parallel, our study showed that OSA patients had significantly reduced S1P levels compared to the healthy control group. In our opinion, the results obtained in this study may indicate dysregulation of the ceramide/S1P rheostat in the course of OSA and a potential reduction in the conversion of ceramide to S1P. Under normal conditions, ceramides are produced acutely via sphingomyelinases (NSmases) in the endothelium and subsequently commonly converted to S1P with the use of ceramidases and sphingosine kinase. Among the ceramide metabolites, S1P is well known to regulate vascular endothelial function by stimulating nitric oxide (NO) production via endothelial nitric oxide synthase (eNOS) (Kerage et al., 2021; SenthilKumar et al., 2024). NO exerts well-documented vasoprotective effects through acute vasodilation, reducing proliferation, migration, thrombosis and inflammation (Su, 2015). However, under chronic pathological conditions when ceramide is produced in excess or endothelial enzymes that metabolize this sphingolipid are impaired, abnormal accumulation of ceramide may occur. Increased cellular levels of ceramide coupled with insufficient conversion to S1P result in the direct activation of protein phosphatase 2A and protein kinase C, of which the former dephosphorylates and inactivates eNOS, while the latter simultaneously phosphorylates and stimulates NADPH oxidase (NOX) to produce reactive oxygen species (ROS) (Zhang et al., 2012; Fox et al., 2007; Cosentino-Gomes et al., 2012; SenthilKumar et al., 2024). Chronically elevated ROS levels result in oxidative stress leading to endothelial dysfunction manifested by increased endothelial permeability, inflammation, and alterations in thrombotic or fibrinolytic mechanisms (Daiber and Chlopicki, 2020). Of note, patients with OSA demonstrate endothelial dysfunction even in the absence of any manifested vascular disease (Ip et al., 2004; Patt et al., 2010).

It is noteworthy that reciprocal changes in plasma ceramide and S1P levels have been observed in patients affected by cardiovascular disease. While S1P is generally reduced in patients affected by CAD (Sattler et al., 2010; Kurano and Yatomi, 2018), those patients affected by familial CAD have higher levels of some ceramide species in plasma compared to healthy controls (Poss et al., 2020). The shift towards ceramides over S1P observed in CAD does not seem surprising, as OSA is one of the strongest risk factors for CAD (Yacoub et al., 2017). Moreover, in CAD, dysfunction and inflammatory activation of the endothelium are pivotal events in the development of atherosclerosis and are associated with an elevated risk of cardiovascular events (Medina-Leyte et al., 2021). There are more examples of disorders connected with OSA in which a dysregulated sphingolipid rheostat may play a role. (i) Lipidomic analysis revealed increased total levels of ceramides alongside very long–chain ceramides in both the myocardium and serum of patients with advanced heart failure (Ji et al., 2017). (ii) A sustained decrease in plasma S1P concentrations in acute myocardial infarction (AMI) patients has been observed. The authors of the study suggested a cardioprotective effect of S1P in AMI, but attributed the most likely reason for S1P reduction to its poor release or increased degradation (Knapp et al., 2013). (iii) S1P levels were significantly lower in patients with pre-diabetes and diabetes mellitus type 2 (T2DM) compared with those without diabetes (Vaisar et al., 2018; Sui et al., 2019). (iv) Patients with ischemic stroke showed lower serum S1P concentrations compared with hemorrhagic stroke patients or healthy controls (Liu et al., 2020).

Despite many reports of the association of both S1P and ceramide with OSA-associated comorbidities, we did not show such a relationship in our study for S1P and ceramide-Ab (as a surrogate for ceramide). This was demonstrated both by bivariate analysis for more frequent comorbidities, such as hypertension, diabetes, and CHD, as well as by using multiple linear regression models that also included COPD and stroke. Increased ceramide-Ab and decreased S1P levels appear to be characteristic of OSA in our patients and not a result of common comorbidities. However, due to the small number of patients with COPD or a history of stroke, the statistical power for these variables may be limited.

Importantly, the results presented in our study regarding S1P are contradictory to the results recently published by Horvath et al. (2023). They noted significantly elevated concentrations of S1P in their OSA patients. The explanation behind this discrepancy is quite puzzling. It may be due to the smaller number of OSA patients (N = 31) and controls (N = 37) recruited by Horvath et al. or a different study design as the authors used a S1P ELISA test from a different manufacturer. Additionally, the medications taken by patients or differences in treatment regimens may play a role. Either way, further studies on representative and well matched cohorts are needed to precisely determine the S1P levels in OSA. Significantly, our study was conducted on larger cohorts (N = 109 and N = 49) and has sufficient power (∼100%) and sample size to detect statistical differences between the tested groups. In addition, our data supports previous reports of lower levels of S1P concentration in patients suffering from comorbidities related to OSA (discussed above).

Interestingly, we observed a trend toward a significant positive correlation between ceramide-Ab levels and the desaturation index. While the correlation is not statistically significant, it suggests the involvement of anti-ceramide antibodies (or ceramide itself) in the pathogenesis of intermittent hypoxia associated with OSA. Moreno et al. (2014) presented a possible explanation for this hypothesis in their publication. They found that ceramide content and ROS production increased in pulmonary arteries (PA) following pulmonary vascular hypoxia. Furthermore, the nSMase inhibitor GW4869 and the anti-ceramide antibody reduced hypoxic pulmonary vasoconstriction (HPV) in chicken PA. The authors concluded that nSMase-derived ceramide could play a critical role in acute oxygen sensing in specialized vascular tissues (Moreno et al., 2014). It is unclear whether the excess anti-ceramide antibodies detected in the serum of OSA patients affect the concentration of active ceramide involved in HPV. Hypothetically, if the antibodies bind to ceramide, they could inhibit pulmonary vasoconstriction induced by intermittent hypoxia. Nevertheless, the question remains as to whether this would constitute a favorable or unfavorable outcome. HPV is an intrinsic homeostatic mechanism of the pulmonary vasculature. In response to alveolar hypoxia, intrapulmonary arteries constrict, diverting blood to better-oxygenated lung segments. This optimizes ventilation/perfusion matching and systemic oxygen delivery (Dunham-Snary et al., 2017). This mechanism, initially protective in nature, becomes detrimental in the chronic setting of OSA, contributing to increased pulmonary artery pressure and vascular remodeling (Balcan et al., 2024). Thus, “switching off” HPV with ceramide antibodies may initially have negative effects, such as disturbing gas exchange in the lungs. However, in the long term, it may prevent pulmonary hypertension and vascular remodeling.

Conversely, anti-ceramide antibodies may block the conversion of ceramide to “protective” S1P, thereby exacerbating the pathological processes in OSA and contributing to endothelial dysfunction (as discussed above). Therefore, we believe it is crucial to investigate this issue in future research.

The literature on the potential physiological role of anti-ceramide antibodies is limited. However, it is known that manufactured ceramide-Abs can bind ceramide and block its functions. For example, Rotolo et al. (2012) demonstrated that ceramide-Ab (2A2) protects against endothelial apoptosis in the small intestinal lamina propria and promotes recovery of crypt stem cell clonogens. This prevented the death of mice from radiation GI syndrome after high radiation doses. Similarly, in a recent publication, Dorweiler et al. (2024) demonstrated that an anti-ceramide antibody is able to reverse the effects of diabetic retinopathy. In this case, ceramide-Ab protects endothelial retinal cells from apoptosis, which is initiated by ceramide induced by TNF-α and IL-1β. Given that elevated levels of ceramide have been well documented in the course of various diseases including cardiovascular, neurodegenerative (Alzheimer’s disease, motor neuron disease), metabolic conditions (obesity, type II diabetes, insulin resistance, impaired glucose tolerance), it is potentially possible to use anti-ceramide antibodies in their diagnosis and treatment (Shen et al. 2025).

Interestingly, in the present study, body mass index was positively correlated with OSA severity (AHI parameter) and with the desaturation index (DI). On the other hand, we detected a negative correlation between BMI and mean SaO_2_ and minimum SaO_2_. Very similar correlations between BMI vs. AHI, DI, as well as mean SaO_2,_ were also recently described by Pau et al. (2023) in Italian OSA patients. These results highlight the very important observation that the higher the BMI, the more severe the episodes of obstruction and desaturation and, consequently, the lower the oxygen saturation during sleep. Positive correlation between BMI and AHI was reported also for patients from China (Liu et al., 2021) and Mauritius (Sant Bakshsingh et al., 2024). Additionally, we found a strong positive correlation between BMI and serum CRP level (r = 0.60). Notably, a similar magnitude of positive correlation between these two parameters was noted by Suša et al., 2021 for Serbian patients (r = 0.633), and a slightly weaker correlation was detected by Guilleminault et al. (2004) (r = 0.459) after adjusting for other coefficients. The correlation between BMI and CRP may be due to the fact that adipose tissue secretes a variety of bioactive mediators including adipocytokines such as adiponectin, leptin, resistin, visfatin or classical cytokines such as tumor necrosis factor α (TNFα), interleukin 1 (IL-1), CC-chemokine ligand 2 (CCL2), and interleukin 6 (IL-6) (Tilg and Moschen, 2006). Among these cytokines, IL-6 is the primary factor driving hepatic CRP production. Importantly, almost one-third of the IL-6 concentration in circulation of obese patients originates from adipose tissue (Mohamed-Ali et al., 1997). Moreover, a significant positive association between IL-6 concentration and fat mass (adipose tissue percentage) was also recently found (Suša et al., 2021).

There are a few limitations of the current study: (1) Testing of anti-ceramide antibodies rather than ceramide itself. Although the measurement of anti-ceramide antibodies is valuable and informative in itself, in the next phase of our research we plan to examine ceramide levels by using the LC-MS/MS technique in our study groups and correlate the obtained results with those obtained from ELISA. Given that two reports (ours and Horváth et al., 2023) are consistent with the elevated levels of anti-ceramide antibodies in OSA patients, we suggest that these antibodies may turn out to be a valuable biomarker for the diagnosis of OSA. This is all the more possible given that increased levels of these antibodies in our patients were not related to presence/absence of comorbidities such as CHD, diabetes, or hypertension. (2) Underrepresentation of women in our control group. Currently, we have a male-to-female ratio of about 6:1, whereas it should be around 3:1 (similar to the patient group). OSA is generally estimated to have a male-to-female ratio of between 3:1 and 5:1 in the general population (Wimms et al., 2016), so we should recruit at least seven more women. However, the gender imbalance in our study did not prevent statistical analysis. (3) Lack of assessment of the concentrations of molecules that bind and transport S1P in the bloodstream, i.e., HDL together with apolipoprotein M or albumin, as their variations may affect the levels of S1P detected in serum.

In conclusion, we demonstrated that anti-ceramide antibody levels were elevated, while S1P concentrations were decreased in patients with obstructive sleep apnea compared to subjects without OSA. Patient BMI was positively correlated with OSA severity, desaturation index, and CRP levels, and negatively correlated with mean SaO_2_ and minimum SaO_2_. We did not find any association of ceramide-Ab and S1P concentrations with the presence of comorbidities such as hypertension, coronary heart disease, diabetes, COPD, and stroke.

## Data Availability

The raw data supporting the conclusions of this article will be made available by the authors, without undue reservation.
